# Supplementary material for: Grapevine Trunk Diseases in Greece: Disease Incidence and Fungi Involved in Discrete Geographical Zones and Varieties
Source: J Fungi (Basel). 2023 Dec 20;10(1):2. doi: 10.3390/jof10010002 (PMC10817465; doi:10.3390/jof10010002)
Supplement: Supplementary file 1 [file jof-10-00002-s001.zip › jof-2758771-supplementary.pdf]

Grapevine trunk diseases in Greece: Disease incidence and fungi involved in discrete geographical zones and varieties

Stefanos I. Testempasis[1], Emmanouil A. Markakis[2,3*], Georgia, I. Tavlaki[2], Stefanos K. Soultatos[2,3], Christos Tsoukas[4], Danai Gkizi[5], Aliki K. Tzima[4], Epameinondas Paplomatas[4], Georgios S. Karaoglanidis[1]

[1] Laboratory of Plant Pathology, School of Agriculture, Faculty of Agriculture, Forestry and Natural Environment, Aristotle University of Thessaloniki, 54124 Thessaloniki, Greece
[2] Laboratory of Mycology, Department of Viticulture, Vegetable Crops, Floriculture and Plant Protection, Institute of Olive Tree, Subtropical Crops and Viticulture, Hellenic Agricultural Organization - DIMITRA, 32[A] Kastorias street, Mesa Katsabas 71307, Heraklion, Crete, Greece
[3] Laboratory of Plant Pathology, Department of Agriculture, School of Agricultural Sciences, Hellenic Mediterranean University, Stavromenos 71004, Heraklion, Crete, Greece
[4] Laboratory of Plant Pathology, Department of Crop Science, School of Plant Sciences, Agricultural University of Athens, Iera Odos 75, Votanikos 11855, Athens, Greece
[5] Department of Wine, Vine and Beverage Sciences, University of West Attica, Ag. Spyridonos 28, 12243, Athens, Greece

*Corresponding author: Emmanouil A. Markakis
E-mail: markakis@elgo.gr

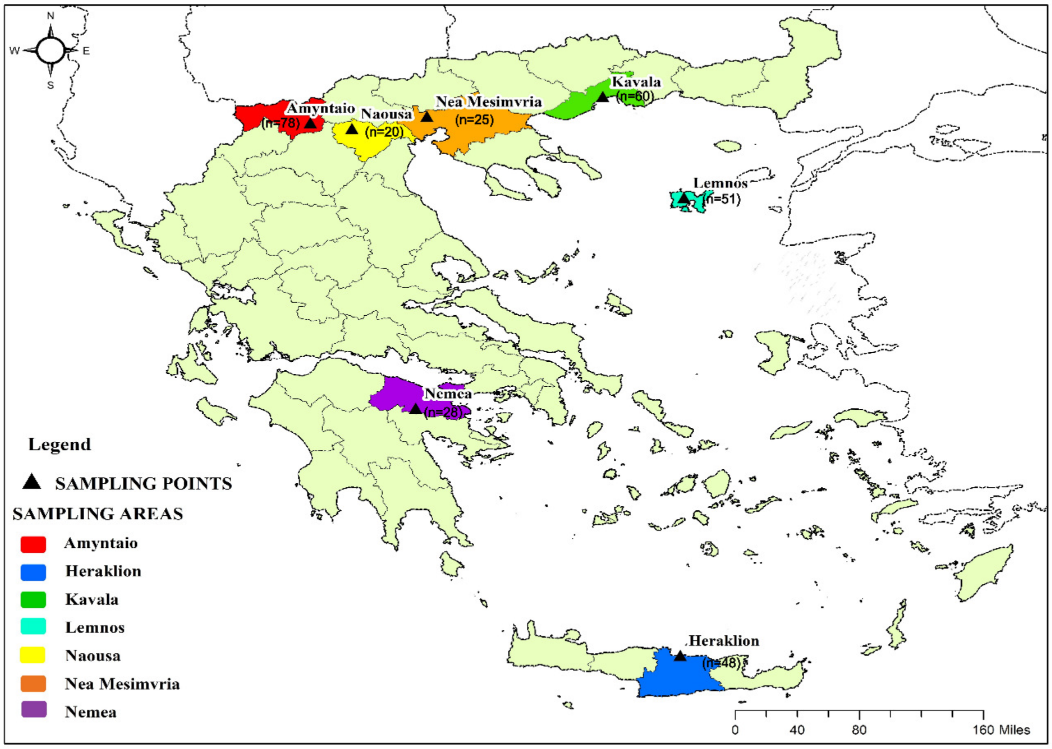

Supplementary Figure S1. Map illustrating the geographical distribution of the surveyed regions across Greece (Northern, Central, and Southern), providing the number of vineyards of the study area's regional coverage.

Supplementary Table S1. Morphological and physiological features of the fungal isolates (mean ± standard error) analyzed in the present study, along with their geographic origin, the plant tissue they were isolated and the GenBank accession numbers determined by the analyses of *internal transcribed spacer regions of ribosomal DNA (rDNA-ITS), rDNA large subunit (LSU), translation elongation factor 1-alpha (tef1-α), β-tubulin (tub2)* and *actin (act)* gene sequences

Isolate[a]	Fungal species[b]	Vine part[c]	Location	Colony color	Growth rate[d] (mm/day)	Conidial dimensions (μm)[e]	Hyphae width (μm)	GenBank accession No.				
								ITS[f]**/Identity (%)**	***LSU***[g]**/Identity (%)**	***tef1-α***[h]**/Identity (%)**	***tub2***[i]**/Identity (%)**	***act***[j]**/Identity (%)**
HOURD2.1AVR1	*Kalmusia variispora*	Cordon	Harakas, Heraklion	White to olive-green, grey	2.97 ± 0.05	Nf	3.10 ± 0.14	OQ683907/100.00%	OQ704313/99.73%	OQ798214/99.67%	OQ784650/99.55%	OQ798208/n.e.
PEROG2.1YP2	*Kalmusia variispora*	Trunk	Asites, Heraklion	White to olive-green, grey	3.95 ± 0.04	Nf	1.99 ± 0.13	OQ683913/100.00%	OQ704319/100.00%	OQ798218/90.46%	OQ784655/99.77%	OQ798213/n.e.
SAROG1.3AVR10	*Seimatosporium vitis*	Cordon	Pretoria, Heraklion	Light brown-black	3.07 ± 0.14	15.29 (±0.38) × 4.73 (±0.12)	2.79 ± 0.18	OQ683910/100.00%	OQ704316/99.39%	OQ798216/100.00%	OQ784653/100.00%	OQ798211/n.e.
SAROG1.3AVR13	*Seimatosporium vitis*	Cordon	Pretoria, Heraklion	Light brown-black	2.16 ± 0.05	17.76 (±0.47) × 5.06 (±0.02)	2.93 ± 0.14	OQ683911/100.00%	OQ704317/99.91%	OQ798217/100.00%	OQ784654/100.00%	OQ798212/n.e.
SAROG1.2AKO1	*Didymosphaeria variabile*	Trunk	Pretoria, Heraklion	White to olive-green, grey	5.61 ± 0.10	Nf	2.90 ± 0.29	OQ683908/100.00%	OQ704314/n.e.	-	OQ784651/99.78%	OQ798209/100.00%
SAROG1.3AVR7	*Neosetophoma italica*	Cordon	Pretoria, Heraklion	White to pale brown	2.61 ± 0.15	7.83 (±0.34) × 3.40 (±0.15)	2.49 ± 0.10	OQ683909/100.00%	OQ704315/n.e.	OQ798215/n.e.	OQ784652/n.e.	OQ798210/n.e.

[a] Codes of fungal isolates obtained from symptomatic grapevine wood.

[b] Fungal species as determined by the analysis of *rDNA-ITS, LSU, tef1-α, tub2* and *act* sequences along with their morphological and physiological features.

[c] Part (cordon or trunk) of vine where fungal strains were isolated on acidified potato dextrose agar (PDA).

[d] Isolates were grown on PDA at 24 °C in the dark and their colonies' diameter was measured at day 3, 7, 10, 14, 17 and 21 or stopped earlier when plate was completely covered by the fungus.

[e] 'nf' indicates that isolates did not form spores within the 21-day incubation period.

[f] Accession numbers of fungal isolates *rDNA-ITS* sequences that yielded and deposited in GenBank.

[g] Accession numbers of fungal isolates *LSU* sequences that yielded and deposited in GenBank. 'n.e.' indicates that no sequence of this fungal species exists in NCBI.

[h] Accession numbers of fungal isolates *tef1-α* sequences that yielded and deposited in GenBank. 'n.e.' indicates that no sequence of this fungal species exists in NCBI.

[i] Accession numbers of fungal isolates *tub2* sequences that yielded and deposited in GenBank. 'n.e.' indicates that no sequence of this fungal species exists in NCBI.

[j] Accession numbers of fungal isolates *act* sequences that yielded and deposited in GenBank. 'n.e.' indicates that no sequence of this fungal species exists in NCBI.

Supplementary Table S2. Sequences used in the phylogenetic analysis of *Neosetophoma*, *Kalmusia*, *Seimatosporium*, and *Didymosphaeria* species.

Species	Culture accession number	Source	Origin	GeneBank accession numbers[a]			
				ITS	LSU	*tef1-a*	*tub2*
Neosetophoma aseptata	CBS 145363	*Viburnum opulus*	Germany	NR_164449	MK540024	NA[1]	NA
Neosetophoma clematidis	MFLUCC 13-0734	*Clematis vitalba*	Italy	NR_154228	KP684153	NA	NA
Neosetophoma shoemakeri	MFLUCC 17-0780	*Malva sp.*	United Kingdom	MG844346	MG844348	NA	NA
Neosetophoma lunariae	CPC 26671	*Lunaria annua*	Germany	NR_154242	KX306789	NA	NA
Neosetophoma salicis	MFLU 17-0118	*Salix sp.*	Uzbekistan	MK608025	MK608026	NA	NA
Neosetophoma poaceicola	MFLUCC 16-0886	*Dead grass*	Thailand	NR_165861	KY550382	NA	NA
Neosetophoma poaceicola	IRAN 2429 C	*Malus domestica*	Iran	KT832078	MT102742	NA	NA
Neosetophoma guiyangensis	GZCC 18-0111	*Dead branch*	China	MH018134	MH018132	NA	NA
Neosetophoma xingrensis	GZCC 18-0110	*Decaying wood*	China	MH018135	MH018133	NA	NA
Neosetophoma italica	**SAROG1.3AVR7**	***Vitis vinifera***	**This study**	**OQ683909**	**OQ704315**	**NA**	**NA**
Neosetophoma italica	MFLU 14-0809	*Iris germanica*	Italy	KP711356	KP711361	NA	NA
Neosetophoma italica	108	*Citrus limon*	Iran	KY290229	KY355076	NA	NA
Neosetophoma garenthjonesii	MFLUCC:14-0528	*Forest soil*	United Kingdom	KY496758	KY496738	NA	NA
Neosetophoma samarorum	CBS 138.96	*Phlox paniculata*	Netherlands	NR_156263	NG_057836	NA	NA
Neosetophoma rosigena	MFLU 17-0626	*Rosa canina*	United Kingdom	NR_157525	NG_059870	NA	NA
Neosetophoma phragmitis	CBS 145364	*Phragmites australis*	Germany	NR_164450	MK540025	NA	NA

Didymella exigua	CBS 183.55	-	France	MH857436	MH868977	NA	NA
Kalmusia variispora	**HOURS2.1AVR1**	***Vitis vinifera***	**This study**	**OQ683907**	**OQ704313**	**NA**	**NA**
Kalmusia variispora	**PEROG2.1YP2**	***Vitis vinifera***	**This study**	**OQ683913**	**OQ704319**	**NA**	**NA**
Kalmusia variispora	KV-9	*Malus domestica*	Iran	OL711708	OL711712	NA	NA
Kalmusia variispora	CBS 121517	-	Syria	JX496030	JX496143	NA	NA
Kalmusia variispora	KV-13	*Malus domestica*	Iran	OL711709	OL711713	NA	NA
Kalmusia italica	MFLU 14-0620	*Spartium junceum*	Italy	KP325440	KP325441	NA	NA
Kalmusia longispora	CBS 582.83	*Arceuthobium pusillum*	Canada	JX496097	JX496210	NA	NA
Kalmusia longispora	T15142	*Vitis vinifera*	Hungary	MN945157	MN945151	NA	NA
Kalmusia longispora	CBS 824.84	*Triticum aestivum*	Germany	JX496115	JX496228	NA	NA
Kalmusia ebuli	CBS 123120	-	-	KF796674	JN644073	NA	NA
Kalmusia cordylines	ZHKU 21-0003	*Cordyline fruticosa*	China	NR_184482	NG_088312	NA	NA
Kalmusia araucariae	CPC 37475	*Araucaria bidwillii*	USA	MT223805	MT223900	NA	NA
Kalmusia spartii	MFLU 14-0751	*Spartium junceum*	Italy	KP744441	KP744487	NA	NA
Helminthosporium leucadendri	CBS 135133	*Carpentaria acuminata*	Australia	KF251150	KF251654	NA	NA
Seimatosporium vitis	Napa764	*Vitis vinifera*	USA	KY706273	KY706298	KY706323	KY706248
Seimatosporium vitis	**SAROG1.3AVR10**	***Vitis vinifera***	**This study**	**OQ683910**	**OQ704316**	**OQ798216**	**OQ784653**
Seimatosporium vitis	**SAROG1.3AVR13**	***Vitis vinifera***	**This study**	**OQ683911**	**OQ704317**	**OQ798217**	**OQ784654**
Seimatosporium vitis-viniferae	L189	*Vitis vinifera*	Cyprus	ON680685	ON692401	ON863781	ON695845
Seimatosporium vitis-viniferae	CBS 123004	*Vitis vinifera*	Spain	MH553992	MH554211	MH554418	MH554660

Seimatosporium vitis-viniferae	P210	*Vitis vinifera*	Cyprus	ON680688	ON692398	ON863780	ON695842
Seimatosporium pistaciae	CBS 138865	*Pistacia vera*	Iran	KP004463	KP004491	MH554432	MH554674
Seimatosporium rosae	CBS 139823	*Rosa sp.*	Russia	LT853105	MH823070	LT853203	LT853253
Seimatosporium germanicum	CBS 437.87	-	Germany	MH554047	MH554259	MH554482	MH554723
Seimatosporium cyprium	CBS 149019	*Vitis vinifera*	Cyprus	ON680684	ON705769	ON863790	ON695856
Seimatosporium sp.	2310C	*Vitis vinifera*	Iran	MW361952.1	MW361960.1	MW375358.1	MW375352.1
Seimatosporium vitisfusiforme	Wint753	*Vitis vinifera*	USA	KY706296.1	KY706321.1	KY706346	KY706271.1
Beltrania rhombica	CBS 123.58	*Mangrove swamp*	Mozambique	MH857718	MH869260	MH704606	MH704631
Didympsphaeria variabile	CBS 120014	*Actinidia chinensis*	Italy	JX496026	JX496139.1	NA	JX496365.1
Didymosphaeria variabile	**SAROG1.2AKO1**	***Vitis vinifera***	**This study**	**OQ683908**	**OQ704314**	**NA**	**OQ784651**
Paraconiothyrium brasilliense	CBS 115.92	*Olea europaea*	Italy	JX496022	JX496135.1	NA	JX496361.1
Paraconiothyrium brasilliense	CBS 587.84	*Vitis vinifera*	Italy	JX496099	JX496212.1	NA	JX496438.1
Paraconiothyrium fuckelii	CBS 508.94	*Rosa sp.*	Netherlands	JX496096	JX496209.1	NA	JX496435.1
Paraconiothyrium fuckelii	CBS 764.71B	*Human*	Netherlands	JX496112	JX496225.1	NA	JX496451.1
Stagonospora paludosa	CBS 135088	*Carex acutiformis*	Netherlands	KF251257	KF251760.1	NA	KF252740.1

[a] NA: Not Applicable

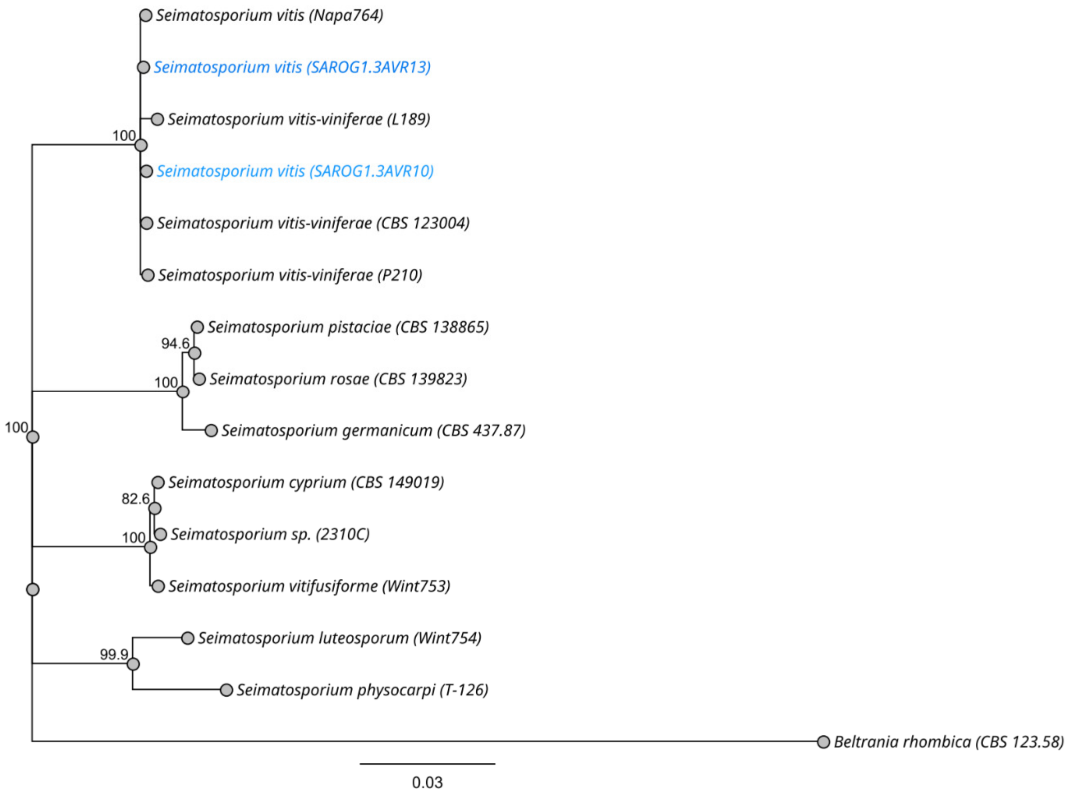

Supplementary Figure S2. Neighbor-joining (NJ) phylogram obtained from the combined *rDNA-ITS*, *LSU*, *tub2*, and *tef1-a* sequence alignment of *Seimatosporium* species. The tree was rooted to *Beltrania rhombica* CBS 123.58. Bootstrap values (1000 replicates) are indicated at the nodes. The scale bar indicated nucleotide substitution in NJ analysis, values ≥ 50% are presented above/below the branches.

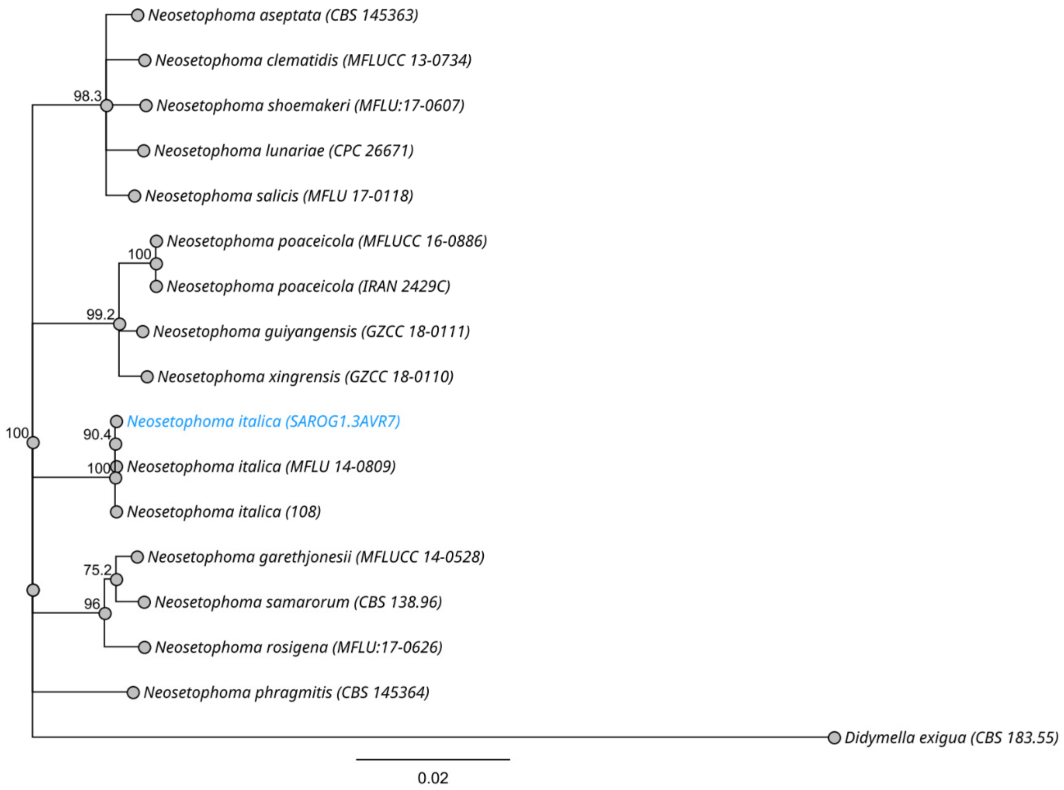

Supplementary Figure S3. Neighbor-joining (NJ) phylogram obtained from the combined *rDNA-ITS, LSU* sequence alignment of *Neosetophoma* species. The tree was rooted to *Didymella exigua* CBS 183.55. Bootstrap values (1000 replicates) are indicated at the nodes. The scale bar indicated nucleotide substitution in NJ analysis, values ≥ 50% are presented above/below the branches.

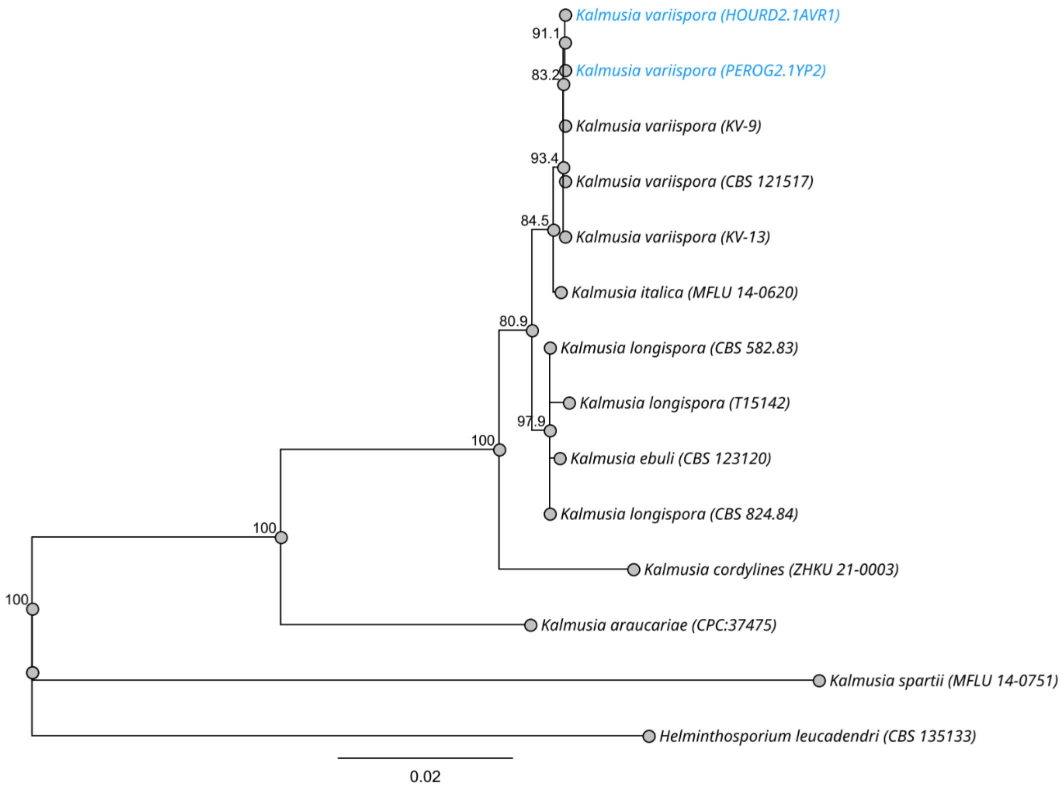

Supplementary Figure S4. Neighbor-joining (NJ) phylogram obtained from the combined *rDNA-ITS, LSU* sequence alignment of *Kalmusia* species. The tree was rooted to *Helminthosporium leucadendri* CBS 135133. Bootstrap values (1000 replicates) are indicated at the nodes. The scale bar indicated nucleotide substitution in NJ analysis, values ≥ 50% are presented above/below the branches.

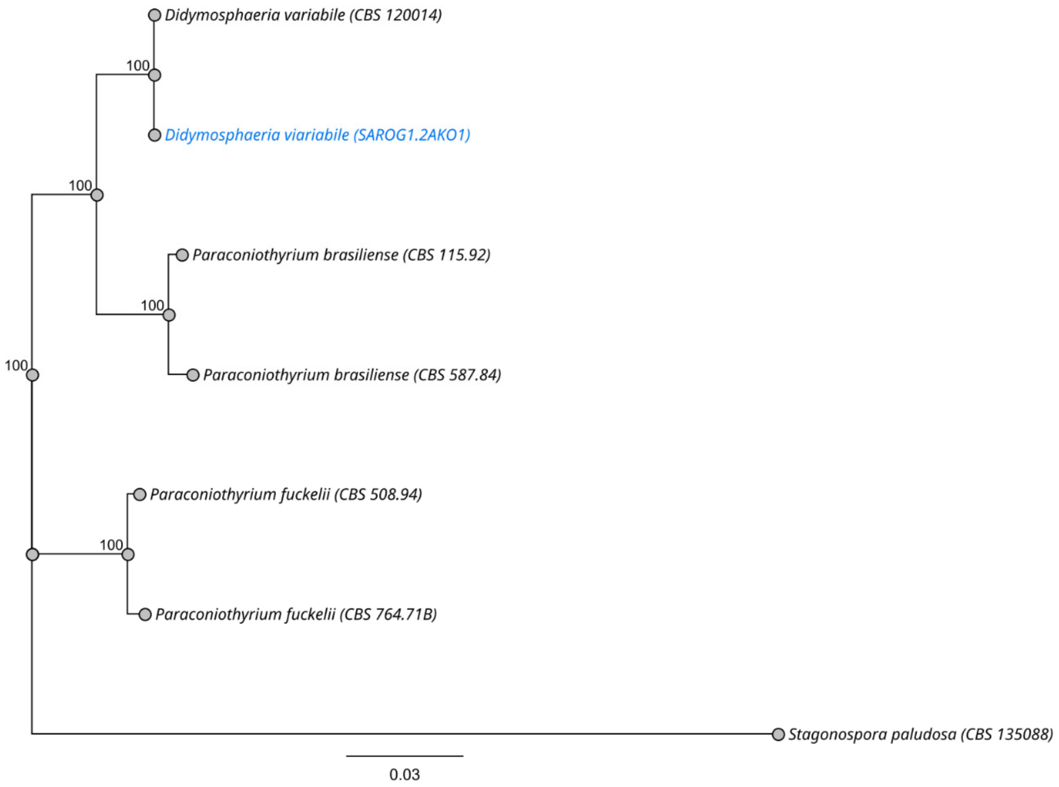

Supplementary Figure S5. Neighbor-joining (NJ) phylogram obtained from the combined rDNA-ITS, LSU, and tub2 sequence alignment of Didymosphaeria and Paraconiothiorum species. The tree was rooted to Stagonospora paludosa CBS 135088. Bootstrap values (1000 replicates) are indicated at the nodes. The scale bar indicated nucleotide substitution in NJ analysis, values ≥ 50% are presented above/below the branches.

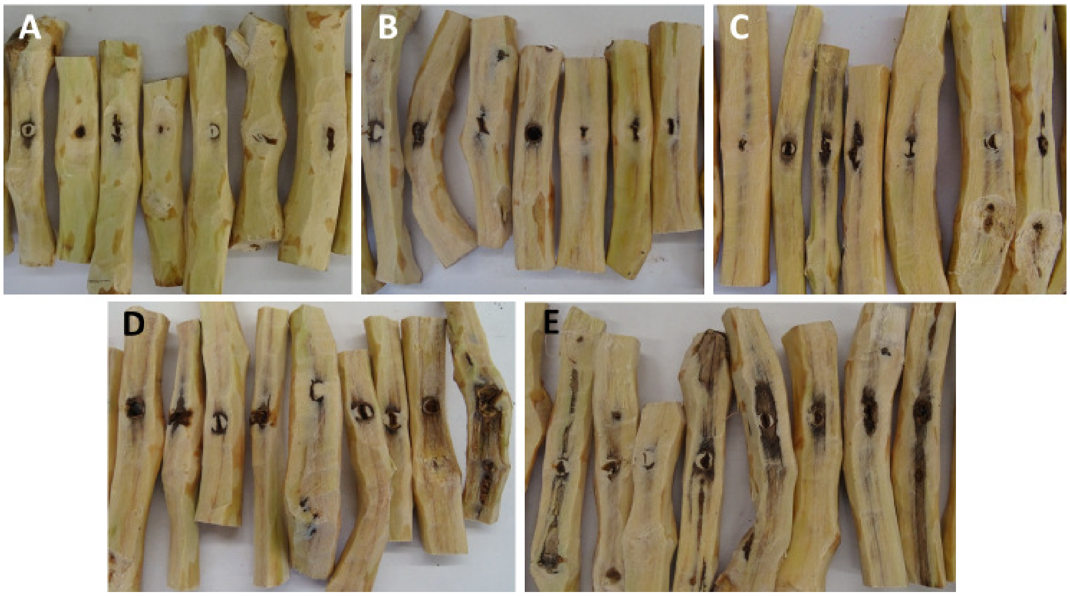

Supplementary Figure S6. Wood discoloration in 2-year-old canes of cv. Soultanina mock-inoculated with sterilized PDA (A) or artificially inoculated with *Seimatosporium vitis* (B), *Didymosphaeria variabile* (C), *Kalmusia variispora* (D) and *Neosetophoma italica* (E), six (6) months post the inoculation.
